# Mental Visualization in the Cerebellum: Rapid Non-motor Learning at Sub-Lobular and Causal Network Levels

**DOI:** 10.3389/fnsys.2021.655514

**Published:** 2021-09-10

**Authors:** Lora T. Likova, Kristyo N. Mineff, Spero C. Nicholas

**Affiliations:** Smith-Kettlewell Eye Research Institute, San Francisco, CA, United States

**Keywords:** mental visualization, cerebellum, plasticity, learning, memory, fMRI, Granger Causal Connectivity

## Abstract

It is generally understood that the main role of the cerebellum is in movement planning and coordination, but neuroimaging has led to striking findings of its involvement in many aspects of cognitive processing. Mental visualization is such a cognitive process, extensively involved in learning and memory, artistic and inventive creativity, etc. Here, our aim was to conduct a multidimensional study of cerebellar involvement in the non-motor cognitive tasks. First, we used fMRI to investigate whether the cognitive task of visualization from an immediate memory of complex spatial structures (line drawings) engages the cerebellum, and identified a cerebellar network of both strongly activated and suppressed regions. Second, the task-specificity of these regions was examined by comparative analysis with the task of perceptual exploration and memorization of the drawings to be later visualized from memory. BOLD response patterns over the iterations of each task differed significantly; unexpectedly, the suppression grew markedly stronger in visualization. Third, to gain insights in the organization of these regions into cerebellar networks, we determined the directed inter-regional causal influences using Granger Causal Connectivity analysis. Additionally, the causal interactions of the cerebellar networks with a large-scale cortical network, the Default Mode Network (DMN), were studied. Fourth, we investigated rapid cognitive learning in the cerebellum at the level of short-term BOLD response evolution within each region of interest, and at the higher level of network reorganization. Our paradigm of interleaved sequences of iteration between two tasks combined with some innovative analyses were instrumental in addressing these questions. In particular, rapid forms of non-motor learning that strongly drive cerebellar plasticity through mental visualization were uncovered and characterized at both sub-lobular and network levels. Collectively, these findings provide novel and expansive insights into high-order cognitive functions in the cerebellum, and its macroscale functional neuroanatomy. They represent a basis for a framework of rapid cerebellar reorganization driven by non-motor learning, with implications for the enhancement of cognitive abilities such as learning and memory.

## Introduction

Traditionally, the cerebellum has been thought to be involved in motor control and coordination (e.g., Evarts and Thach, 1969; Gilbert and Thach, 1977; Brooks and Thach, 2011). Over the last years, increasing functional Magnetic Resonance Imaging (fMRI) evidence has been accumulating that the cerebellum is contributing to a wide range of cognitive functions (Ito, 1984; Llinas, 1985; Kelly and Strick, 2003; Buckner et al., 2011; Diedrichsen and Zotow, 2015; D’Angelo and Casali, 2013; Brissenden et al., 2018; King et al., 2019; Schmahmann et al., 2019). Working memory, language, executive function and affective tasks have been shown to elicit largely non-overlapping patterns of activation within cerebellar cortex (Allen et al., 1997; Kelly and Strick, 2003; Chen and Desmond, 2005). Additionally, functional connectivity studies in humans (Stoodley and Schmahmann, 2009; Buckner et al., 2011) have shown that cerebellar regions communicate with non-motor networks of the cerebral cortex, manifesting a coarse functional organization unsuspected until fairly recently. These findings of a significant role of the cerebellum in functions traditionally reserved for the cerebral cortex are consistent with recent estimates of its large area, now understood to be close to 80% of the area of the cerebral cortex (Sereno et al., 2020).

### Cerebellar Macroscale Functional Anatomy

*Motor vs. non-motor cerebellar lobules.* Macroscale cerebellar neuroscience is already well-developed in a large body of work (e.g., Habas et al., 2009; Stoodley and Schmahmann, 2009, Stoodley et al., 2010, 2012; O’Reilly et al., 2010; Buckner et al., 2011; Keren-Happuch et al., 2014; Van Overwalle et al., 2014; Brissenden et al., 2016; Guell et al., 2018a, b; Marek et al., 2018; King et al., 2019; Schmahmann et al., 2019; Guell and Schmahmann, 2020). As a result, “the cerebellum is now appreciated as a structure relevant for virtually all aspects of behavior in health and disease” (Van Overwalle et al., 2014).

**FIGURE 1:**
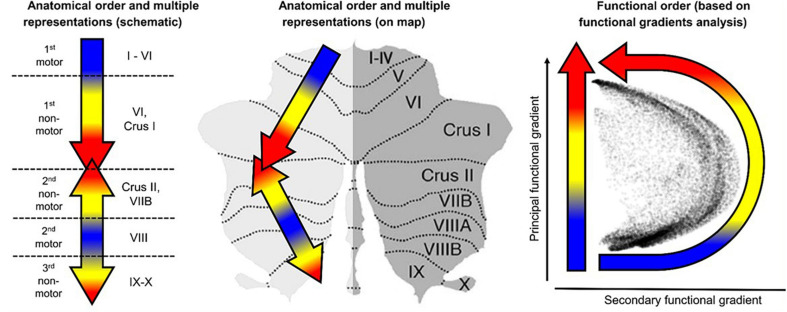
Didactic summary of cerebellar functional anatomy based on human fMRI evidence. Data-driven analyses of cerebellar fMRI indicate three major fundamental poles of cerebellar functional neuroanatomy. (**Left,Center**) Motor processing (blue) is represented twice in each cerebellar cortical hemisphere (lobules I–VI; lobule VIII). Non-motor processes (red and yellow) are represented three times in each cerebellar cortical hemisphere (lobules VI-Crus I; lobules Crus II–VIIB; lobules IX–X). A specific anatomical order is conserved throughout the cerebellar cortex, and propagates from first motor toward first non-motor representation (i.e., from lobules I–VI to Crus I), from second motor toward second non-motor representation (i.e., from lobule VIII to Crus II), and from second motor toward third non-motor representation (i.e., from lobule VIII to lobule IX/X). (**Right**) The principal axis of macroscale functional organization in the cerebellar cortex progresses from motor, to attentional/executive, to default-mode processing. This progression is captured in the anatomical order of cerebellar functional territories as shown in the center and right panels, and also revealed by a data-driven analysis of functional gradients in the cerebellar cortex based on resting-state functional connectivity between cerebellar cortical areas (After Guell and Schmahmann, 2020; reproduced with permission).

A didactic summary on the *cerebellar macroscale functional anatomy and organizational principles* derived from numerous functional imaging studies that have mapped motor and non-motor task processes, as well resting-state networks in the human cerebellum, was recently published by Guell and Schmahmann (2020). These principles include the existence of multiple areas of both motor and non-motor representations, with a specific ordering of the functional domains (motor and non-motor), which “together define the position of, and relationship between, each functional territory in cerebellar macroscale functional anatomy.” Their data-driven analysis of functional gradient (see Figure 1) revealed that *motor* processing is represented twice in each cerebellar cortical hemisphere, though limited to lobules I–VI, and lobule VIII (with a focus on VIIIa), while *non-motor* processes are represented three times - in lobules VI-Crus I, in lobules Crus II–VIIB, and in lobules IX–X (see also Snider and Eldred, 1952; Buckner et al., 2011; Guell et al., 2018a, b).

### Cerebellar Functional Boundaries and Organization

The finer-scale *functional boundaries* in the human cerebellum have also been actively investigated (e.g., Diedrichsen and Zotow, 2015; King et al., 2019). In particular, King et al. (2019) used a *multi-domain task battery* to assess a *broad range of cognitive processes*. A battery of 26 diverse tasks comprising 47 unique conditions, was run over four sessions in 1,000 subjects using intrinsic functional connectivity MRI (fcMRI), allowing them to derive a comprehensive functional parcelation of the cerebellar cortex, which was further evaluated by predicting functional boundaries in a novel set of tasks. The organization of a variety of *both motor and non-motor* subdivisions have been also estimated by other methods, such as intrinsic functional connectivity (e.g., Yeo et al., 2011; Buckner et al., 2011).

### Rationale and Overview of the Study Design

**FIGURE 2:**
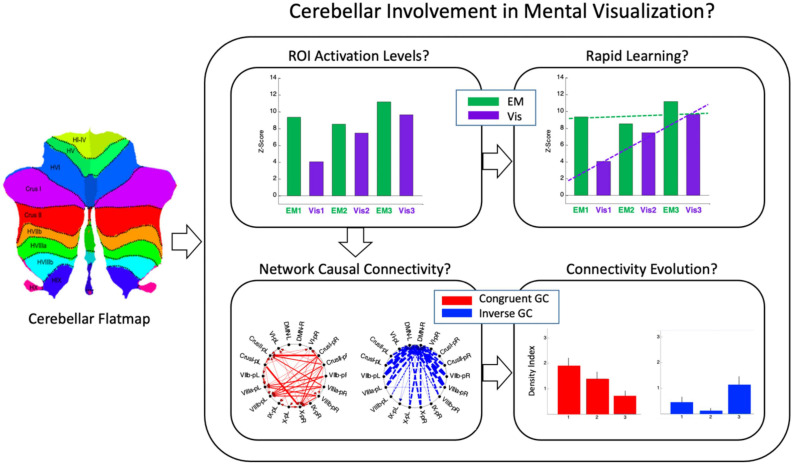
A schematic preview that shows at glance the overall structure of the methodological design. Average cerebellar activation was mapped to an unfolded cerebellar flatmap (icon at left) where the pattern of cerebellar involvement in non-motor cognitive tasks was used to determine the activation levels in each local region of interest (ROI), and its rapid change over three repeats of each task (*top row*); Granger Causal connectivity was assessed within the respective networks of these ROIs, and the connectivity evolution over the three repeats plotted in terms of a Connectivity Density Index (*bottom row*). Two interleaved tasks: ***EM*** = *Explore and Memorize*; ***Vis*** = *Visualization from memory* (see Figure 3 in Experimental Design).

Here, we perform a multidimensional fMRI feasibility study of the involvement of the cerebellum in the cognitive task of visualization from an immediately acquired memory of complex spatial structures (line drawings). The study is designed to probe the involvement of the human cerebellum in the task of mental visualization, and examine the task-specificity of the cerebellum regions involved by a comparative analyses with the task of perceptual exploration and memorization of the drawings that had to be later visualized from memory (see overview in Figure 2). Granger Causal Connectivity analysis (GCCA) is used to determine the directed causal influences among the cerebellar network nodes; furthermore, the causal interaction of the cerebellar networks with a key large-scale cerebral cortical network, such as the Default Mode Network (DMN) are determined as well.

Moreover, this study is also designed to evaluate a number of questions of the role of the cerebellum in *learning*. Does *rapid learning-driven reorganization* occur in the cerebellum during visualization-from-memory iterated on a short-time scale? As the learning proceeds, what types of reorganization take place at sub-lobular and at network level in the cerebellum? What large-scale cerebral networks do they couple/uncouple to/from as a function of this rapid learning? And furthermore, how do these processes differ during the visualization phases relative to the perceptual viewing/memorization phases of the same images?

While cognitive functions, such as visual working memory, are well established within the cerebellum, to the best of our knowledge, the special class of (spatial) memory visualization and furthermore, its involvement in learning, have not been previously studied. The current results include the finding of a *well-structured cerebellar network* for visualization, cerebellum-based *rapid learning effects in this high-order cognitive task* at both the local sub-lobular and the network levels, and strong *causal cerebellar-cerebral* interactions. These results provide novel insights into the significant role of the cerebellum in cognition, in particular in the cognitive processes of learning and memory, and its macroscale functional organization.

## Materials and Methods

### Participants

The participants were volunteers with normal or corrected-to-normal visual acuity (5 male, 2 female; ages 34-70). The experimental protocol was approved by the Smith-Kettlewell Institutional Review Board; prior to participating, all volunteers provided their informed consent. Participants were compensated for their time. All procedures were conducted in conformity to the Declaration of Helsinki.

### Experimental Design

**FIGURE 3:**
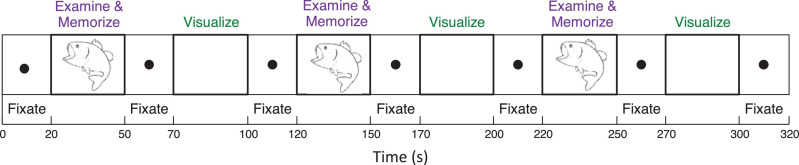
Functional Magnetic Resonance Imaging experimental design included a sequence of two tasks (i) perceptual exploration and memorization of complex graphic material (30 s) and (ii) visualization-from-memory of these images (30 s), interleaved with fixation periods (20 s); this sequence was repeated three times for each image.

To analyze mechanisms of visualization from immediate memory and rapid learning, sighted adults performed a sequence of alternating (i) *Explore and Memorize* (***EM***): perceptual exploration and memorization of complex graphic material (30 s) and (ii) *Visualization-from-Memory* (***Vis***): visualization of these images from memory of the prior exposure in ***EM*** (30 s), interleaved with fixation periods (20 s); this sequence was repeated three times for each image in a 3T Siemens Prisma scanner (see Figure 3). The images were scientific and artistic line drawings.

### MRI Data Collection, Analysis, and Visualization

#### SUIT: Cerebellum-Specific Anatomical Processing

**FIGURE 4:**
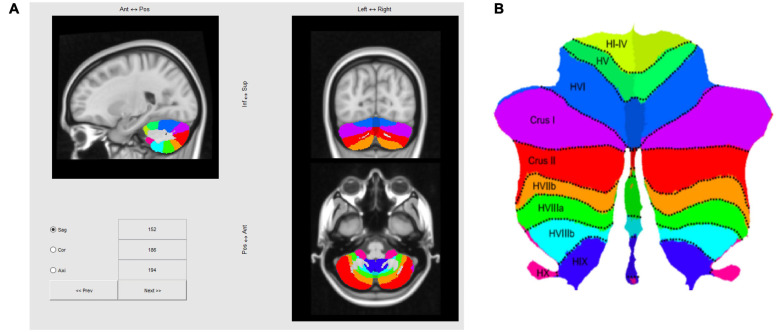
**(A)** Three-slice volume view of the maximum probability atlas transformed by SUIT to the MNI152 T1 MRI scan used as the structural reference for registering multi-subject fMRI data in this study. **(B)** Probabilistic atlas of the cerebellar lobules projected to a flatmap of the cerebellum from the SUIT Matlab toolbox (Diedrichsen and Zotow, 2015; http://www.diedrichsenlab.org/imaging/suit_flatmap.htm).

The cerebellum was segmented using the SUIT Matlab toolbox developed by the Diedrichsen Lab (^1^ Diedrichsen, 2006; Diedrichsen et al., 2009, 2011; Diedrichsen and Zotow, 2015; King et al., 2019), based on a T1 anatomical scan after reconstruction by Freesurfer^2^, which yielded a 1 mm isotropic resolution. This is the structural volume to which our fMRI data are registered, so once this volume is processed with SUIT, fMRI activation maps could be transformed to a standard flattened representation of the cerebellum, as depicted in Figure 4. Note that in the text positions of regions of interest (ROIs) will be referenced in the rostral/caudal and medial/lateral framework of the flatmap, e.g., those in lobules such as V, or VI will be referred as “rostral” relative to lobules such as VIII, IX, or X, which will be labeled as “caudal.”

#### Data Acquisition and Pre-processing

##### Functional Magnetic Resonance Imaging Acquisition

MR data were collected on a 3T Siemens Prisma Fit magnet equipped with a 64-channel head + neck coil. BOLD responses were obtained using an EPI acquisition (TR = 2 s, TE = 30 ms, flip angle = 45, voxel size = 2.5 × 2.5 × 2.5) consisting of 54 axial slices extending across the whole brain. Pre-processing was conducted using FSL (Analysis Group, FMRIB, Oxford, United Kingdom) and included slice-time correction and two-phase motion correction, consisting of both within-scan and between-scan 6-parameter rigid-body corrections. No spatial smoothing of the 3D fMRI data was imposed. Activation maps were averaged across the depth of cerebellar gray matter in 20% increments from white to pial surface models to generate the data shown on the 2D representations of the flattened cerebellum.

To facilitate segmentation and registration, a whole-brain high-resolution T1-weighted anatomical scan was also obtained for each participant (voxel size = 0.8 × 0.8 × 0.8 mm). White matter segmentation in this T1 scan was conducted using FreeSurfer and gray matter was generated with the mrGray function in the mrVista software package^3^.

##### Functional Magnetic Resonance Imaging Time-Course Analyses

The data were averaged from the individual participant brains into the Montreal Neurological Institute average of 305 individuals^4^. The data were analyzed with the Stanford VISTA Lab software. The effective neural activation amplitudes (e.g., Friston et al., 1994) for each task across the repeats of multi-task sequences in the 1.5 h scan were estimated by the following procedure. A General Linear Model (GLM) consisting of boxcar neural task activations and an auditory stimulus regressor was convolved with an estimated hemodynamic response function (HRF) and fitted to the blood-oxygen-level-dependent (BOLD) responses along with a 4th order polynomial to remove baseline trends. BOLD amplitudes were defined as “task-positive” or “task-negative” according to the sign of the GLM beta fit.

##### Regions of Interest Activation Analysis

Regions of interest (ROIs) were generated for each of the visualization task-positive and task-negative BOLD activation regions in the cerebellum; the Talairach locations specified in Table 1. The cerebral Default Mode Network (DMN) ROI was taken from Yeo et al. (2011) 7-network cortical parcelation, and converted from a Freesurfer average subject to the MNI base anatomy used in this project. The effective neural activation amplitudes for each condition in each defined ROI was estimated by the same GLM procedure applied to the average signal across all voxels within the ROI.

**TABLE 1:** Talairach Locations of the Cerebellar ROIs.

**ROI name**	***X* Tal**	***Y* Tal**	***Z* Tal**
VI-pL	−22	−67	−25
VI-pR	23	−58	−24
Crusl-pL	−38	−62	−31
Crusl-pR	35	−56	−29
Crusll-pL	−40	−63	−48
Crusll-pR	17	−70	−40
Vllb-pL	−31	−60	−49
Vllb-pR	27	−61	−48
Vllla-pL	−22	−54	−49
Vllla-pR	28	−9	−49
Vlllb-pL	−17	−47	−45
Vlllb-pR	19	−42	−45
IX-pL	−14	−46	−44
IX-pR	12	−47	−44
X-pL	−23	−34	−38
X-pR	21	−31	−38
Crusl-nL	−36	−68	−32
Crusl-nR	33	−69	−32
Vl-vermis-n	0	−63	−24
Crusll-nL	−24	−74	−37
Crusll-nR	24	−74	−36
IX-nL	−7	−49	−41
IX-nR	5	−47	−44

*ROI, region of interest; Tal, Talairach coordinates; VI, VIIb, VIIIa/b, IX, X, CrusI, Crus II, cerebellar lobules; n, negative ROI; p, positive ROI; L, left hemisphere; R, right hemisphere.*

#### Rapid Learning Slope Analysis

The rapid learning effect as expressed by changes in the strength of the BOLD signal across repeats for each task were analyzed by calculating the slope of a linear regression of the z-score of the GLM amplitude for each of the 3 repeats of a task. ROIs that were classified as “task-negative” had the sign of their responses inverted prior to slope calculations. Slopes were normalized to the mean absolute value of z-score of the 3 repeats, so that they represent fractional changes relative to the mean.

#### Granger Causality Analysis Procedures

These analyses followed the procedures described in Cacciamani and Likova (2017). Starting from seed ROIs, Granger causality maps were generated in two directions, from the seed to every voxel in the brain (x to y), and from every voxel to the seed region (y to x), for each of three tasks (PE, MD, S) during the fMRI scan cycle. First, 50-s temporal segments of BOLD data were extracted starting at the onset of each 30-s task through the end of the 20-s rest interval following the task (in order to account for any task-related functional connectivity effects that may persist into this interval). The 2-s repetition time (TR) gave were 25 BOLD volumes in these segments, from which the average time course of all voxels that were members of the seed ROI was computed. Multiple linear regressions were performed to fit the 2nd-25th volumes of each voxel in the brain as a function of the 1st-24th volumes of themselves and as a function of the seed ROI, plus a constant term (x to y), and analogously for the y tox direction. Regression coefficients between the ROI and these individual voxels were converted to z-scores by dividing by the estimated standard error of the coefficient. Those with z-scores sufficiently different from zero implied a causal linkage (Granger, 1969), with the sign of the z-score indicating whether the causality was congruent with or inverse to the source signal (see section “Results” for details).

For pairwise ROI waveform Granger causality analyses, the “full” model containing the prior time points both ROIs was compared to a “reduced” model containing only the prior time point of the ROI being modeled. The variance ratio of reduced and full models was tested against a null hypothesis of 1, with *p*-Values coming from the F distribution. The *p*-Values were converted into z-scores based on the standard normal distribution in some analyses for ease of interpretation.

#### Rapid Connectivity Reorganization

The time course of the connectivity reorganization was accessed by the *density* of causal connections in a network of ROIs where the pairwise *p*-Value of Granger Causality as described above was below a specified threshold. *Connection density* among a particular network of ROIs was defined as the number of connections divided by the total number of ROIs in the network.

## Results

### Cerebellar Network for Visualization From Memory

**FIGURE 5:**
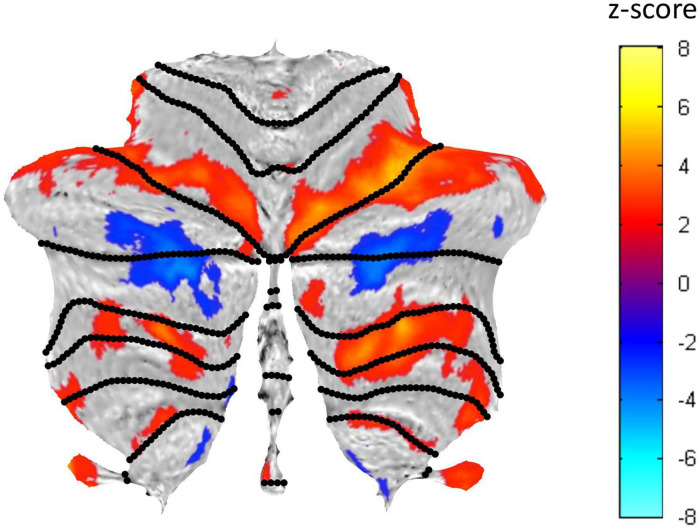
Cerebellar flatmap representation of the average activation across the three Visualization repeats, thresholded at z-score of ±0.5. Positive BOLD activation coded in warm colors and negative BOLD activation in cool colors. The regions of activated voxels within each lobule were used to define the ROIs for further analysis.

**FIGURE 6:**
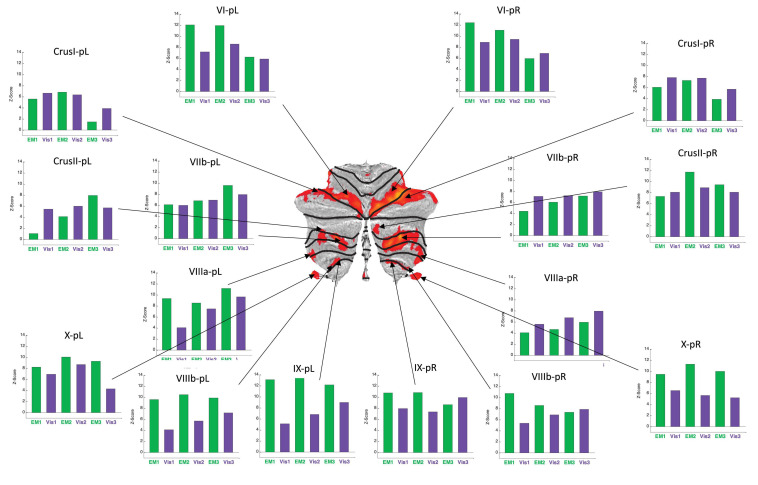
Activation patterns for the positive cerebellar ROIs identified in the present protocol. *Visualization* (***Vis***) and *Explore and Memorize* (***EM****)* task periods are coded by the **green** and **purple** bars, respectively. Note that the activation strengths in both tasks was generally similar for left and right hemisphere in the most rostral and caudal ROIs (in the flatmap framework). Note that the responses are plotted as z-scores. For this sample, z-scores of >2.45 are significant at *p* < 0.05 (two-tailed) and z-scores >5.95 are significant at *p* < 0.001 (two-tailed), etc. All specified ROIs reach the higher criterion for significance in at least one repeat in each of the tasks.

**FIGURE 7:**
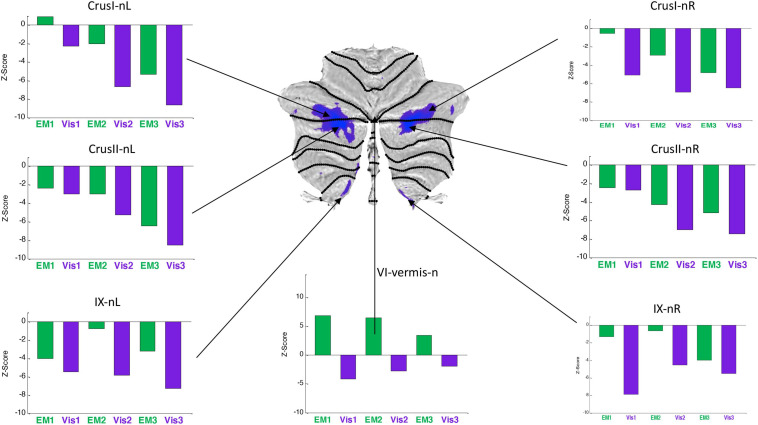
Activation patterns for the *task-negative* cerebellar lobular ROIs, expressed as z-scores in the same format, and to the same statistical criteria, as for Figure 6. Note that, the responses were negative for both *Visualization* (***Vis***) and *Explore and Memorize* (***EM***) tasks, and tended to become increasingly negative over the three repeats in Crus 1 and Crus II bilaterally. To the significance criteria specified in Figure 6, responses for *Vis* in all specified negative ROIs reach the higher criterion for significance in at least one repeat, except for the VI-vermis-n ROI, where ***EM*** reached this criterion. Lobule IX showed a hemispherically asymmetric pattern in both tasks, as opposed to the symmetric pattern in Crus I/II, and to the predominant case for the task-positive ***Vis*** ROIs of Figure 4. The VI-vermis-n ROI produced weak negative ***Vis*** responses, but in contrast the rest of these ROIs, its ***EM*** responses inverted to positive activation; in general, both ***Vis*** and ***EM*** responses became significantly more negative over the repeats.

A well-structured visualization network of activated and suppressed BOLD regions was identified in the cerebellum (Figure 5). This visualization network involves both *task-positive* (see Figure 6) and *task-negative* (see Figure 7) regions. Their BOLD response characteristics were analyzed as described in the following sections.

### Average GLM Cerebellar Activation Patterns

The average BOLD activation signal for each ROI was analyzed by the standard general linear model (GLM) approach, with a separate regressor for each of the six 30 s task periods (see Materials and Methods). The z-scores of the average BOLD response from the GLM, averaged across the participants, are shown as the bar graphs placed around the respective cerebellar flatmap (see Figures 6, 7). The green bars code is for the Exploration and Memorization (***EM***) epochs, and purple bars - for the Visualization (***Vis***) epochs. A z-score range of ±1 corresponds to the standard error of the z-score means.

BOLD activation patterns for the task-positive cerebellar ROIs identified in the present protocol are shown in Figure 6, averaged over the battery of images used in the study. Note that in most cases the engaged ROIs exhibited bilaterally symmetric response patterns. These responses exhibited significant changes as function of task repetitions, showing the tendency to significantly increase over the repeats in some ROIs, or to decrease in others. Importantly, in many cases these tendencies were different for the ***Vis*** and the ***EM*** task sequences within a particular ROI, showing that this learning manifests task-specificity, as is further analyzed below. These tendencies represent a *rapid evolution* of the cerebellar response strengths with task repetitions, reflecting plastic reorganization in cerebellar involvement over this short time scale as a form of rapid learning.

Average response patterns for the cerebellar ROIs suppressed in ***Vis*** are shown in Figure 7. The responses of all of these ROIs - except that of lobule VI-vermis-n - were also negative in the ***EM*** task. Note that the strength of the response in most ROIs systematically changes over the three task repetitions. In particular, the suppressed Crus I and Crus II ROIs responses tend to become increasingly negative over the repeats in both the ***Vis*** and ***EM*** tasks. ***Vis*** generated significantly stronger responses than ***EM*** in all task-negative ROIs (with the VI-vermis being the only exception).

### Analysis of Rapid Learning Effects

**FIGURE 8:**
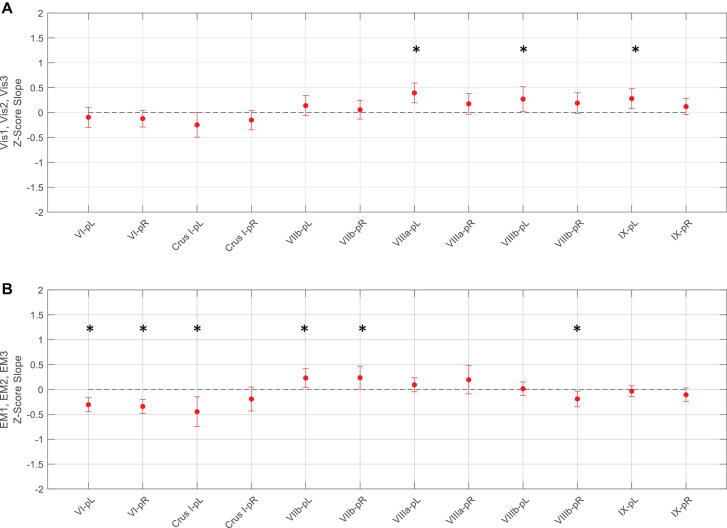
Slopes of the evolution of average response strengths (red dots) for the task-positive ROIs from Figure 5 as a function of task repeats for the ***Vis* task** (**upper panel A**) and ***EM* task** (**lower panel B**). Note that for the ***Vis*** task sequence **(A)**, three ROIs in the left lobules VIIIa, VIIIb, and IX had significant positive slopes - increasing response strength as the learning progressed. In contrast, the ***EM*** task sequence **(B)**, showed both significant decreases and increases in different ROIs during learning. Error bars are 95% confidence intervals for the difference of the slopes from a zero-slope; asterisks indicate significant slopes at *p* < 0.05.

**FIGURE 9:**
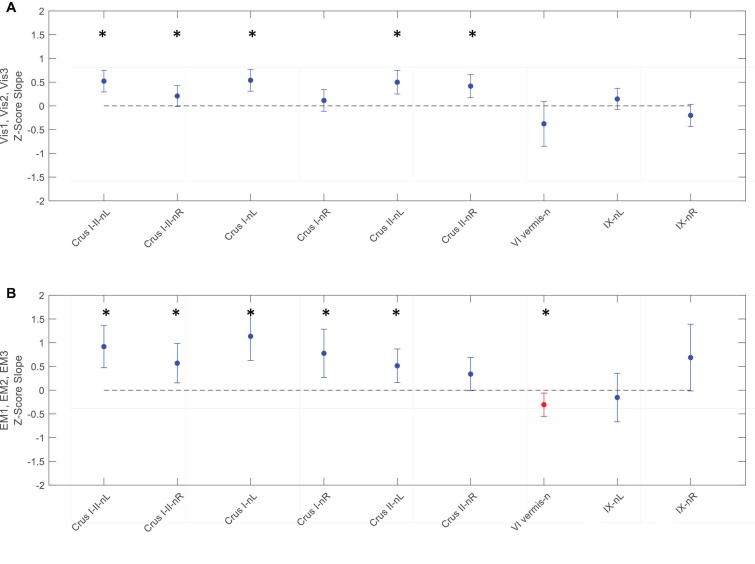
Slopes of the evolution of response strengths (blue dots) for the task-negative ROIs from Figure 6, with the same format and significance criteria as for Figure 8. One ROI had positive activation (red dot) for the ***EM*** task **(B)**. Note that most of the task-negative Crus ROIs had significant slopes for the ***Vis* task** sequence **(A)**, with increasing negative response strength as the learning proceeded, thus deepening the suppression in these regions. A similar picture, with even greater suppression, is seen for the ***EM* task** sequence **(B)**. Asterisks indicate significant slopes at *p* < 0.05.

To investigate the temporal evolution of the rapid learning effects seen in Figures 6, 7, we analyzed the change in activation across the perceptual exploration and memorization, ***EM*** and across the visualization, ***Vis*** repeats in each region through a formal *slope analysis* (see Methods) separately for each task, and plotted in Figures 8, 9 below the slopes of the change of response strengths as a function of task repeats for the sets of ROIs from Figures 6, 7, respectively. Slopes are coded in terms of ***absolute*** BOLD response strength, of either negative or positive sign, so that an increasing slope for a *task-negative ROI* implies that the BOLD response strength is becoming increasingly negative.

For the ***Vis*** task sequence, the ***task-positive* ROIs** in the left lobules VIIIa, VIIIb, and IX had significant slopes (Figure 8), implying that they had increasing response strength as the learning proceeded. Note that for the Vis task sequence (Figure 8A), three ROIs in the left lobules VIIIa, VIIIb, and IX had significant positive slopes – increasing response strength as the learning progressed. In contrast, in the EM task sequence (Figure 8B), the first three (rostral) ROIs – in lobules VI left/right, and Crus I, left - showed significant decreases, i.e., negative slopes, as did the most caudal ROI (VIIIb, right), indicating reduced involvement with learning. Two ROIs, on the other hand – VIIb L/R – showed significant increases during the EM repeats, implying increased cognitive involvement during learning.

In contrast to the task-positive ROIs, the slope analysis in the *task-negative* ROIs revealed stronger bilateral learning effects for both ***Vis*** and ***EM*** tasks. Slopes of the evolution of response strengths for these ROIs are shown in Figure 9. There were significant slopes for the large task-negative (Crus I and Crus II) ROIs for the ***Vis*** task, implying increase of the suppression strength in these regions as the learning proceeded. A similar reorganization pattern was seen for the ***EM*** task sequence. Note that short-term learning effects more complex than a first-order assessment (i.e., than linear slopes) are beyond the scope of this analysis.

### Granger Causal Connectivity Analysis

It is important to have clear terminology for the respective polarities of the BOLD activity in the source brain region, the polarity of the causal influence, and the polarity of the effect in the recipient brain region. Thus, for the BOLD activity, we will use the terms “positive BOLD” (or “activation”) for an increase from the resting level, and “negative BOLD” (or “suppression”) for a decrease from the resting level. Visualization task-positive and visualization task-negative networks are analyzed in Section “Granger Causal Connectivity Analysis for the Task-Positive Network” and “Granger Causal Connectivity Analysis of the Task-Negative Network,” respectively (Note that “suppression” is used here in the sense of a relative reduction, not absolute elimination).

For causal influences, we will use the term “*congruent* causal influences” for those that provide a positive correlation with the source activity, and “*inverse* causal influences” for those that provide a negative correlation with the source activity. The rationale for this terminology is that the source signal could be either positive or negative BOLD, so that a congruent influence will itself be positive or negative, respectively, according to the sign of the source signal, while an inverse influence will be the converse (negative for a positive source, and vice versa). Moreover, the effects of these influences could correspondingly be facilitatory or inhibitory according to the sign of the BOLD signal in the recipient brain region.

#### Granger Causal Connectivity Analysis for the Task-Positive Network

##### Visualization From Short-Term Memory

**FIGURE 10:**
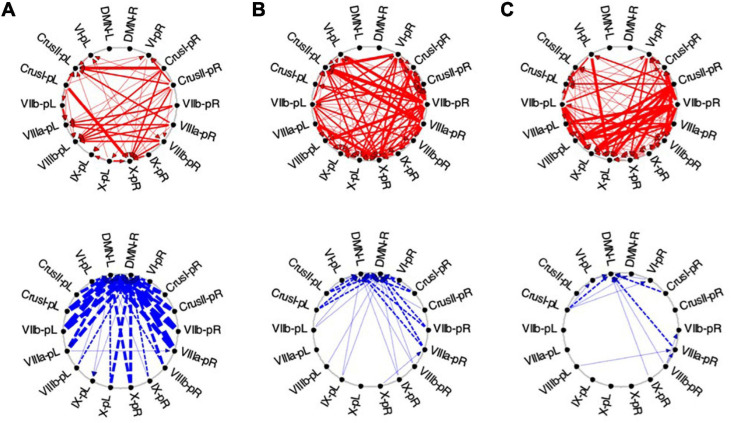
Average Granger causal connectivity in *Visualization* (*Vis*) for the *task-positive cerebellar ROIs* with each other and with the *cortical DMN*. Connections are shown as arrowed lines if the directed connectivity is significant at *p* **<** 0.05, with line thickness coding connectivity strength, and red/blue color coding whether the influence is congruent or inverse relative to the BOLD signal (in separate upper and lower plots). **(A–C)**: Granger causal connectivity for *Vis 1*, *2, 3* (the first through third visualization repeats). There is no congruent connectivity to the DMN in this task-positive ROIs, while the dominant inverse connectivity flows from almost all of these ROIs to the DMN initially, decreasing dramatically with task repeats.

Average Granger Causal connectivity was analyzed separately for each repeat during the *Visualization* task sequence, and was followed by an innovative analysis to assess if there was a rapid learning reorganization in the connectivity over the task repeats. The directed Granger connectivity for the task-positive cerebellar ROIs of Figure 6 with each other, and with the left and right hemisphere ROIs for the Default Mode Network (DMN) (which has been shown to have strong connections with Crus I/II of the cerebellum; e.g., Buckner, 2013), are shown in circular plots in Figure 10.

For the first Visualization repeat (***Vis 1***), the congruent causal influences were primarily directed from many cerebellar ROIs to Crus II and lobule VIIIa/b ROIs on the left, and to the lobule X ROI on the right side, with no connections either to or from the cortical DMN (Figure 10A, upper row). The inverse causal connectivity for these ROIs had a dramatically inverted pattern, with virtually all cerebellar ROIs sending inverse influences to the cortical DMN ROIs bilaterally, and none inversely influencing each other (Figure 10A, bottom row); both the density and the strength of these decreased dramatically over the repetitions.

In the second Visualization period (***Vis 2***) the directed connectivity was similar to that for ***Vis 1***, but with notable modulations (Figure 10B). The intra-cerebellar *congruent* connectivity increased significantly relative to ***Vis 1***, with the main congruent influences being from the lobule VIIIa and VIIb ROIs to the rest of the cerebellar ROIs (Figure 10B, upper row). The cortical DMN ROIs again remained unconnected in either direction. The *inverse* connectivity generally replicated the pattern to that for ***Vis 1*** repeat, but with notably reduced strength throughout (Figure 10B, lower row).

In the last Visualization repeat (***Vis 3***) the *congruent* directed connectivity retained the general bilateral pattern of ***Vis 2*** (Figure 10C), but with progressive differentiation between the caudal cerebellar ROIs, whose intra-cerebellar influences shifted to involve bilateral lobules VIIb-L/R, VIIIa-R influencing the more caudal ROIs (VIIIb and IX), and lobule VI-L/R strongly influencing lobule IX; while the rostral ROIs VI and Crus I-left, received fewer and weaker influences from the caudal ones. The cortical DMN ROIs again remained unconnected in either direction (Figure 10C, upper). The *inverse* connectivity, on the other hand, further weakened from its pattern in ***Vis 2*** (Figure 10C, lower).

##### Exploration and Memorization

**FIGURE 11:**
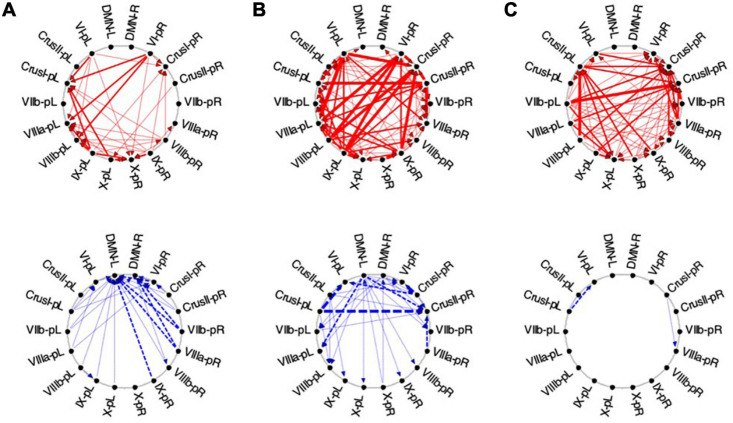
Average Granger Causal connectivity for the perceptual *exploration and Memorization* **(*EM*)** task in the *task-positive* network; shown with the same format and significance criteria as for Figure 10. The connectivity patterns had some general similarities to that for the *Vis* task in Figure 10, but also major differences, in that the dominant inverse connectivity flowed from the DMN to these ROIs in the second repeat.

In the task-positive network, the lack of any involvement of the cortical DMN ROIs represents the main general similarity of the directed ***congruent*** causal influences for ***EM*** to that for ***Vis*** (Figure 11). For ***EM 1***, the congruent causal influences are directed mainly to lobule VIIIa/b on the left side, bilateral Crus 1 and right lobule X (Figure 11A, upper), with the most rostral and caudal lobules - VI and bilateral IX - being the main sources of these causal influences. For the *inverse causal influences* from/to the ***Vis 1*** ROIs, the pattern is a weaker version of the inverted pattern for ***Vis 1***, with most causal influences directed from the cerebellar ROIs toward the left DMN ROI (Figure 11A, lower).

In ***EM1***, Crus I bilaterally, lobule VIIIb left, and X right were the main hubs of converging *congruent* influences, the DMN not either impacting or being impacted by *congruent* cerebellar influences, however, DMN was the target of all *inverse* causal influences.

As was the case for Vis 2, the congruent causal connectivity for EM 2 strengthened relative to EM 1, but now primarily between mid-caudal to rostral ROIs (VIIIa/b, VI and Crus I), with the cortical DMN ROIs remaining almost entirely disconnected (Figure 11, upper row). The inverse causal influences, however (with respect both to EM 1 and to Vis 2), predominantly headed from the DMN ROIs back to the Crus I and lobule VIIIa/b ROIs (Figure 11, lower row).

By the third EM period, the pattern reorganized again, with the intra-cerebellar congruent influences now focusing on the right hemisphere ROIs, though still failing to connect with the cortical DMN ROIs at all (Figure 11B, upper). The inverse influences almost entirely dropped away (Figure 11B, lower).

#### Granger Causal Connectivity Analysis of the Task-Negative Network

##### Visualization

**FIGURE 12:**
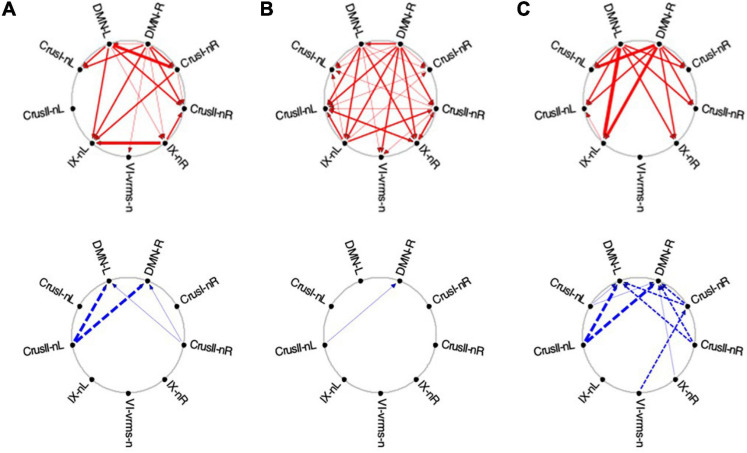
Average Granger causal connectivity for *Visualization* in the *task-negative network* (shown in the same format and significance criteria as for Figure 10). Upper row: Congruent *causal influences* within the network of cerebellar ROIs and the cortical DMN. Lower row: Inverse *causal influences*. The Granger Causal Connectivity demonstrates rapid network reorganization over the *visualization* task repeats. The DMN in strongly involved bilaterally, sending congruent GC influences to these task-negative ROIs during all three repeats, and receiving weak inverse influences from the Crus ROIs.

**FIGURE 13:**
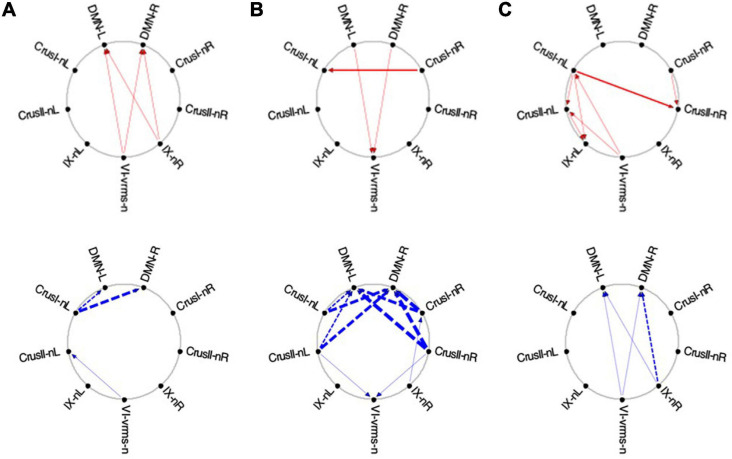
Average Granger causal connectivity maps in the *EM* condition for the task-negative network (shown in the same format and significance criteria as Figure 10). The congruent influences were dramatically weaker than for the visualization task, while inverse influences were reorganized. Influences flow inconsistently from and to the DM at different times, but strongly to the DMN from the Crus ROIs for inverse influences during the second repeat.

The average Granger causal connectivity for the *task-negative* network during visualization are shown in Figures 12, 13, but it should be reiterated that the *congruent* causal connectivity (red arrows) now represents a negative influence on the recipient ROI, since it is deriving from a negative (or suppressive) BOLD signal. The *inverse* causal connectivity (blue arrows), on the other hand, represents an inversion of the negative influence of the source ROI, and hence a positive signal at the recipient ROI.

Initially, the *congruent* drive for the task-negative network mainly flowed from DMN to the Crus I, Crus II-nR and lobule IX ROIs (Figure 12A, upper row), with a reciprocal influence back from Crus I-nR to DMN-R. *Inverse* influences fed back from Crus II to the DMN ROIs (Figure 12A, lower). In ***Vis 2***, the *congruent* drive from the cortical DMN ROIs strengthened (Figure 12B, upper row). *Inverse* influences were almost absent for these task-negative ROIs (Figure 12B, lower). Remarkably, however, by ***Vis 3***, the pattern of reciprocal connectivity focused entirely strong *congruent* influences flowing bilaterally from the cortical DMN ROIs to all the cerebellar ROIs in the network (Figure 12C, upper row), while reciprocal *inverse* influences from Crus I/II flowed back to DMN (Figure 12C, lower row).

##### Exploration and Memorization

Finally, for the ***EM*** task, its task-negative network showed reduced connectivity as a whole. Across all task repeats, the *congruent* influence in ***EM 1*** was dramatically weaker (Figures 13A-C, upper) than was seen in the ***Vis*** conditions in Figure 10. The *inverse* influences were somewhat stronger, heading back to the DMN ROIs (Figures 13A-C, lower). Notably, in ***EM 2***, the *congruent* DMN influences have largely evaporated (Figure 13B, upper), whereas strong *inverse* influences now head from both Crus I and Crus II bilaterally to the DMN ROIs (Figure 13B, lower). This pattern closely resembles that in ***Vis 3*** above (Figure 11C, lower row). By ***EM 3***, the *congruent* influences have disappeared, with some Crus I, Crus II and IX interconnectivity appearing (Figure 13C, upper), and reciprocal *inverse* influences run from Crus II back to the DMN (Figure 13C, lower).

### Evolution of the GC Connectivity

**FIGURE 14:**
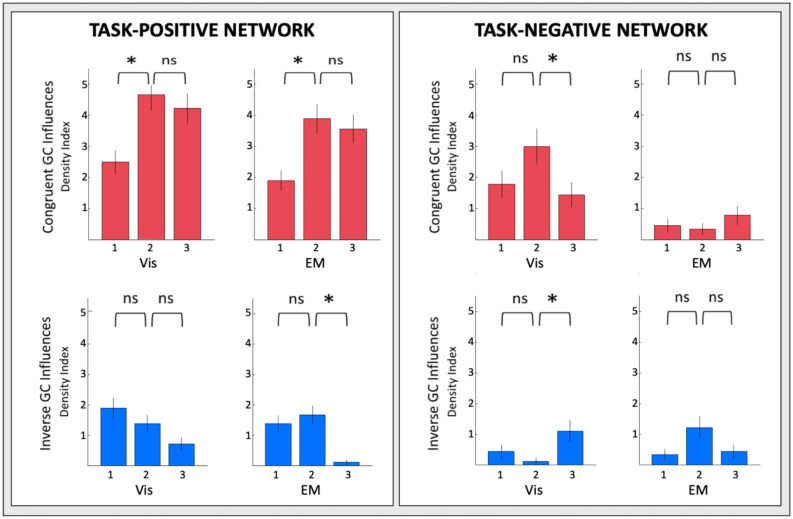
Overview of the density of connections under each circular plot of Figures 9–12. Error bars are 1 SEM of the counts. Sequential pairwise differences significant at *p* **<** 0.05 are indicated by asterisks.

The changes of the GC connectivity pattern over task repeats were quantified in terms of ***connectivity density***, or the number of significant connections relative to the number of ROIs in the task-positive or task-negative networks in each condition. A summary overview of the numbers of significant GC influences in each condition is provided in Figure 14. For the *task-positive* network, the *congruent* GC influences (Figure 14, upper left plots) increased significantly from ***Vis 1*** to ***Vis 2***, then does not change significantly. The same pattern of ceiling in the third repeat is seen for the ***EM*** conditions. The *inverse* influences (Figure 14, lower left plots) showed a contrary pattern, decreasing almost linearly with repeat number, and significantly so from both ***Vis 1*** to ***Vis 2*** and ***EM 1*** to ***EM 2***.

For the *task-negative* network, on the other hand (Figure 14, righthand plots), the GC influences in general are significantly weaker than those for the ***Vis 1*** ROIs. The *congruent* influences in the visualization task (Figure 14, upper right plots) show an inverted-V pattern: they first increased from ***Vis 1*** to ***Vis 2*** and then decreased from ***Vis 2*** to ***Vis 3***, while there was no significant change in the very few ***EM*** congruent influences. On the other hand, the *inverse* influences in the ***Vis*** repeats (Figure 14, lower right plots), though only marginally significant, seemed to start low but to increase at the third ***Vis*** repeat, while the ***EM*** influences appeared to follow a weak form of an inverted-V function, peaking at the second repeat ***EM 2*** and then falling back again at ***EM 3***.

## Discussion

Our aim was multidimensional. First, we investigated whether the entirely cognitive, no-motor-response task of visualization from an immediately acquired memory of complex spatial structures (line drawings), engages the cerebellum. This investigation led to the establishment of a respective, wide-spread cerebellar network. Second, we examined the level of task-specificity of the cerebellum regions involved by running comparative analyses with the perceptual exploration and memorization of the drawings that had to be later visualized from memory. Third, new insights about the cerebellar network organization were gained by Granger Causal Connectivity analysis, which determined the directed causal influences among the network nodes; furthermore, the causal interaction of the cerebellar networks with a key large-scale cortical network, such as the DMN was studied as well. Fourth, rapid learning effects on both the BOLD response reorganization in each region of interest, as well as at the higher – network – level, were investigated. Taken together, this array of analyses provides novel and expansive insights into the entirely cognitive functions, such as mental visualization from non-motor memory in the cerebellum, and its macroscale functional neuroanatomy. To our best knowledge, this is the first study of cerebellum reorganization on the very short time scale of immediate non-motor learning through visualization.

### Visualization-From-Memory in the Cerebellum

#### Overview

##### Cerebellar Macroscale Organization

First, the results will be interpreted in the basic *framework of macroscale organizational principles of the cerebellum*, developed by Guell and Schmahmann (2020), which summarizes a wide range of data on cerebellar activation patterns into a scheme of *motor* and *non-motor* cerebellar function (Figure 1). This scheme shows that cerebellar lobules 1-VI and VIII are involved in motor processing of various kinds, while lobule VI/Crus I, Crus II, VIIB and IX-X are involved in diverse aspects of cognitive (non-motor) processing.

##### Cerebellar Functional Boundaries

Second, the results are further discussed in the context of *cerebellar functional boundaries and organization*, based on the landmark multidimensional investigations of cognitive and motor functions in the cerebellum (Diedrichsen and Zotow, 2015; King et al., 2019). To characterize the functional diversity of the cerebellum, and determine if it is organized into *distinct functional subregions*, they ran a Multi-Domain Task Battery (MDTB) battery of 47 unique conditions, thus providing an extensive atlas of cerebellar function to which the present results can be related.

##### Cerebellar Flatmap

The relative locations of the involved lobular and sub-lobular ROIs are referenced in the context of the cerebellar flatmap used for functional atlases.

As a whole, the analysis showed that memory visualization powerfully engages a network of positive and negative sub-lobular regions in the human cerebellum, and drives rapid learning at both local and network levels.

#### Visualization-Positive Network

First, in terms of the cerebellar *macroscale organization*, the *task-positive* visualization network (Figure 6) encompassed regions across all three *non-motor* territories (lobules VI-Crus I; lobules Crus II–VIIB; and lobules IX–X), plus - to some degree – the *“second motor* representation” (lobules VIII) of Guell and Schmahmann (2020) (Figure 1; but see, e.g., Brissenden et al., 2018, and King et al., 2019, below for the involvement of these lobules in non-motor functions as well). Notably, the largest positive visualization clusters were in the *non-motor* regions along most of lobule VI/Crus I border bilaterally, and in lobule VIIb. The clusters in lobule VIIIb gravitated to the borders with its neighbors – lobules IX and VIIIa. Well-structured clusters, in particular, followed the transitional region along lobules VIIIb/IX border bilaterally. Remarkably, the whole of lobule X was activated bilaterally – a rare occurrence in other fMRI studies of this tonsillar, which is usually only partially activated (e.g., Diedrichsen and Zotow, 2015; King et al., 2019).

Second, from the perspective of *functional boundaries* or the *cognitive descriptors* for the *functional regions* in the MDTB parcelation (King et al., 2019^5^), the complex pattern of the *positively only* activated network (Figure 6) in the present *visualization-from-immediate-memory* task, had some commonalities with the *“active maintenance” and “working memory”* patterns of the MDTB. In particular, the lobule VI/Crus I border and lobule VIIb ROIs activated in *visualization* are also involved in *active maintenance*: *working memory* paradigms have been previously found to activate a constellation of lobule VI, Crus I, and lobule VIIIa (Stoodley et al., 2012); a functional dissociation between both visual working memory and visuospatial attentional processing has been found within VIIb/VIIIa (Brissenden et al., 2018); and the nearby lobule VIIIb/IX border bilaterally is the main region activated in “*spatial imagery*” (King et al., 2019). The upper part of lobule X is implicated in tasks such as “*visual working memory*” and “*saccades*;” but again, note that uniquely, this lobule was fully activated in the current study.

This comparative analysis of the regions in the visualization-positive network is in line with a view of *cerebellar computational modules*, flexibly reconfigured as components in the architecture of different tasks.

#### Visualization-Negative Network

The *visualization-negative* network (Figure 7) exhibited a bilaterally symmetric pattern located entirely within *non-motor* (see Figure 1) cerebellar territories. The largest clusters run across the central section of the Crus I/Crus II border, accompanied by suppression of the most medial part of lobule IX.

First, it is interesting to find that, in the context of Guell and Schmahmann (2020) *macroscale functional anatomy* of the cerebellum (Figure 1), our task-negative visualization network (Figure 7), closely resembles a constellation of Crus I, II and lobule IX ROIs representing the cerebellar component of the *Default Mode Network (DMN)*. Consistent with this finding, the Granger Causal Connectivity analysis of cerebrum/cerebellar interactions revealed that, the DMN is the one that causally drives the suppression in the whole *Visualization-negative network* (Figure 12, top row).

Second, the results were further examined in the context of the *functional boundaries* delineated by the comprehensive Multi-Domain Task Battery flatmaps (King et al., 2019; see text footnote 5). A core component of our visualization-from-memory task is working memory (WM). Key modules of the Baddeley model of WM are the *visuospatial sketchpad* (pictorial information) and the *phonological loop* (verbal, phonological information), which are proposed to store information separately in memory. Further, the *episodic buffer* integrates these two types of information for more effective transfer from and to long-term memory (Baddeley, 1992, 2003; Lohr and Gall, 2008).

In terms of this model, our visualization of *visuo-spatial, pictorial* stimuli, accompanied by neither spoken nor written verbal information, and requiring no response (neither motor nor non-motor) to be planned or executed, should engage the *visuospatial sketchpad*, not the phonological loop. Moreover, note that the task of recall in our experiment is also spatial/pictorial – the participants have to “*see*” the explored images on their *mental “sketchpad,”* not to name or recognize them in any other form.

In spite of our heavily visuo-spatial/pictorial paradigm, is it still possible that some covert verbal form of processing, such as (covert) naming, may explain the results? According to the Dual-Coding Theory proposed by Allan Paivio in 1971, both visual and verbal information can be used to enhance the storage and recall of information (e.g., Paivio, 1971, 1986) (There are, however, limitations of the dual-coding theory and alternative theories, such as the propositional and the common coding theories.) Marvel and Desmond (2010) proposed that “the cerebellum enhances working memory by supporting inner speech mechanisms,” and that it is tied to verbal working memory. This concept is elaborated in the Marvel et al. (2019) review, which emphasizes that this motor system support includes cerebral regions that are involved in motor planning and preparation, together with their cerebellar counterparts. Such motor planning and preparation activities further include decision making, attentional and choice activities, which are generally considered to be cognitive processes, although specific to the motor response domain.

Our study was not designed to test hypothetical contribution of any inner/covert verbal mechanisms, so we can only speculate on this issue. First, as summarized above, the experiment does not involve any (overt) motor or verbal stimulus component, preparation or execution task, and maximally isolates any potential covert form of these. In principle, it is possible to have covert naming but if that was happening, it would occupy only a negligibly small segment of the 30 sec long visualization phases, and would not explain the strong prolonged activation in non-verbal working memory sub-lobular regions, such as along the VI-Cruz I border or lobule VIIb.

Second, previous studies have found strong lateralization of cerebellar activation during speech activity. Our visualization response pattern, on the other hand, is almost entirely bilaterally symmetric, implying that it is not mediated by any form of verbal response.

Third, more detailed analysis in the terms of the cognitive descriptors for the ten functional subdivisions in the MDTB parcelation, where each is described by the three features that best characterize it (King et al., 2019; see text footnote 5), shows that the negative signal that we found in Crus I/II, in particular, represents *suppression* of the functional subdivisions for narrative event sequence network, such as based on a story telling vs. math subtraction, and language processing (left hemisphere), and suppression of word comprehension, verbal fluency, narrative, word comprehension and language processing (right hemisphere). Importantly, this finding implies that the task of visualization of memorized spatial/pictorial information may need, and even benefit from, the active *suppression* of competing systems of a linguistic nature.

In summary, though there could be some covert motor component, such as inner speech, underlying activation in cognitive tasks, this is not found for the present task. As reviewed above, there is a systematic distinction between the cerebellar lobules engaged in cognitive tasks vs. those involved in motor tasks (e.g., Guell and Schmahmann, 2020). Furthermore, large test battery studies, such as the 47-conditions in King et al. (2019), demonstrate a broad variety of distinct activation patterns for a diverse array of cognitive tasks, implying that the mechanisms engaging the cerebellum cannot be put under the one common denominator of motor activity, be it overt or covert. Our analysis above, in particular, the finding of *active suppression* of the language related areas in the cognitive task of visualization of pictorial information, provides further support for this position.

#### The Pattern of Cerebellar Activation/Suppression

A possible expectation could be that the visualization activation/suppression pattern as a whole would resemble that of tasks such as *spatial imagery*, *object working memory*, or even mental manipulations such as *mental rotation*. This was not the case, however (see the functional atlas of King et al., 2019). The visualization pattern differed significantly from that for each of these tasks. There was some partial overlap, such as with a few regions of the (2-back) working memory task, however, the differences were dramatic.

Interestingly, the visualization activation/suppression pattern closely approximated the spatial map network in that atlas. What is in common between the *Visualization* and the *spatial map* tasks is that both deal with spatial structures. The complex line-drawing stimuli recalled *during Visualization*, can be put in the larger context of the representation of spatial maps. However, our *Visualization* task more closely corresponds to that of “*subsequent recall*,” which is not what they measured. Beyond the similarity of the *Visualization* and “spatial map” activation patterns, there are differences. One main difference was found in VI-vermis, which is strongly activated in the “*spatial map*” task, as opposed to being *suppressed* in *Visualization*. The two tasks also differently engage lobule X - there was only a partial activation in “spatial map” vs. full activation in *Visualization*.

Thus, the present analysis shows that the task of *visualization-from-immediate-memory* engages a different and far more elaborate network of functional cerebellar regions than the previously studied tasks of similar categories.

### Rapid Learning-Driven Cerebellar Reorganization in a Cognitive Task

#### Cerebellar Region Level: BOLD Response Changes in Each ROI

The slope analysis we developed for identifying a (first-order) systematic increase/decrease in response strength over the three repeats of the learning sequence revealed significant rapid learning changes in both ***Vis*** and ***EM***.

For the ***Vis*** task sequence, three *task*-*positive* ROIs in left lobules VIIIa/b and IX had a significant slope (Figure 8), implying that it had increasing response strength as the learning proceeded. Interestingly, a rapid learning effect, expressed as significantly negative slopes, was broadly present in the ***EM*** task sequence (three rostral and the most caudal right positive ROIs) indicating reduced engagement with learning. This reduction may be interpreted as either task optimization or reduction of attention as the image became progressively more familiar through the processes of learning and memorization.

In contrast to the task-positive visualization ROIs, the slope analysis in the *task-negative* ROIs revealed stronger bilateral learning effects for both ***Vis*** and ***EM*** (Figure 9). In particular, positive slopes in the DMN-connected regions Crus I and II implied increase of the suppression strength in these regions as the learning proceeded. The reorganization pattern in the ***EM*** task sequence had similar characteristics.

#### Cerebellar Network Level: Rapid Reorganization of Causal Connectivity

The widespread activation in all sectors of the cerebellum in these visualization and perceptual/memorization tasks supports the extensive reports of cerebellar involvement in a variety of forms of cognitive processing (see above).

To investigate how these multiple cerebellar regions interact with each other, we used Granger Causal (GC) Connectivity analysis, which allowed us to establish not only the presence of an interaction but to determine its causal nature and direction. Furthermore, our examination was not limited to the “congruent” (or positively correlated) causal influences, as is most often done, but included the “inverse” (or negatively correlated) ones as well (see Figures 10–13).

In term of GC influences between the cerebral Default Mode Network and the cerebellar networks for the ***Vis*** and ***EM*** tasks, a remarkable result was that the DMN did not exercise any GC influence on any task-positive cerebellar ROI, but only on the task-negative ROIs Crus I, Crus II and lobule IX bilaterally. DMN received, however, inverse GC influences from both positive and negative cerebellar ROIs (Figures 10–13).

Furthermore, it was fascinating to uncover *rapid learning reorganization* even at the *network level* of causal influence. ROI-specific connections, their strength and even their directions were changing markedly over the learning repeats. To quantitatively capture some global effects, we developed a “*Density Index*,” which reflects the “extent of communication,” or proportion of GC influences, within each network. Significant rapid connectivity reorganization, as expressed by the Density Index, was found in the *task-positive network*, with similar Density Index profiles in ***Vis*** and ***EM*** for both their congruent and inverse GC connections: (i) the density of *congruent* causal interactions ramped up highly significantly from the first to the second repeat, with this increase staying sustained in the third repeat; (ii) the density of the *inverse* ones declined with repeats. On the other hand, the *task-negative network* showed a variety of density changes, with the stronger effect on its congruent ***Vis*** GC influences in an inverted-U form, picking up in the second repeat (see Figure 14).

#### Time-Scale of Reorganization

The present paradigm was targeted to explore possible reorganization in a cognitive task, and furthermore, to explore it on the *short time-scale* of a typical fMRI scan, instead of a time-scale of days or months. Thus, this study allowed us to “zoom in” to observe temporal evolution happening over just a few minutes, driven by the process of *cognitive* task repetition.

With respect to longer-term evolution, we have previously investigated the effect of 5 days of 2 h/day of specialized training - the Cognitive-Kinesthetic memory-drawing training in the blind (Likova, 2015). The fMRI assessments were run at *three time points*: (1) before starting the training, (2) immediately on completing the week’s training, and (3) again after a two-month consolidation period without further training. The results revealed a remarkable temporal sequence of training-based brain reorganization in both the hippocampal complex and the temporal-lobe object-processing hierarchy just after the training, with the reorganization continuing to evolve over the prolonged consolidation period. These changes were not just statistically significant, but were often of the same order of magnitude as the activations themselves.

For example, a hippocampal pattern of profound learning-based transformations was strongly reflected in the primary visual cortex V1, with the retrieval function showing massive growth during blind memory-drawing as result of the Cognitive-Kinesthetic training and consolidation, while the initially strong hippocampal response during tactile exploration and memory encoding disappeared. Furthermore, after training, a cascade of discrete ventral regions in the form of an alternating local patch structure underwent radical transformations to reach complete functional specialization for either encoding or retrieval, implying a complex reorganization of the object processing sub-networks through the learning period. These results showed, for the first time, such a *learning-based* reorganization in the form of a *posterior-anterior cascade of* functionally *dissociated patches.* This novel finding of a multifold learning-based reorganization of the “temporal stream” is consistent with the model of the ventral stream as incorporating a number of recursive subnetworks (Kravitz et al., 2013), rather than being just a simple feedforward pathway.

In support of profound reorganization on the same timescale, a recent study (Stevenson et al., 2021) has shown that a similar duration (4-days) of training in the *motor* domain of acrobatic tasks increased both Purkinje-cell synaptogenesis and astrocytic volume in the rat cerebellum relative to a control group. Such cerebellar changes happening over only a *few days* were considered *“rapid”* in comparison to the *months* of typical motor training effects. In this context, the changes on the scale of minutes that we found in the current study as a result of a series of repetitions during fMRI scanning may have to be designated as *ultra-rapid*.

## Conclusion

Overall, the results revealed a well-structured cerebellar network for *visualization from memory* for the first time. This network involved both activated and suppressed regions. Surprisingly, the generally overlapping *perception/memorization* network did not evoke the consistently stronger activation that might have been expected. All suppressive responses, in particular, were markedly stronger in visualization.

Remarkable rapid reorganization was observed in the response patterns over the task iterations in most cerebellar activation sites. In terms of the temporal evolution of the rapid learning process, some cerebellar sites showed significantly increasing activation as the learning through the task sequence progressed, while others showed significantly increasing suppression, revealing a progressive differentiation of the cerebellar responses. These effects were *task-specific*.

At a network level, both congruent and inverse causal connectivity influences were identified between non-motor cerebellar regions. Furthermore, our paradigm of interleaved sequences of task repeats revealed an (ultra) rapid reorganization in the cerebellar connectivity relationships as well as in their individual response strengths discussed above.

These multidimensional fMRI and connectivity findings provide a solid basis for a novel framework for the investigation of rapid cerebellar and cerebellar-cerebral reorganization during non-motor cognitive tasks. To our knowledge, this study is the first to reveal this form of (ultra) rapid non-motor/cognitive learning and neuroplasticity in the cerebellum, as well as being the first to investigate this process both at a sub-lobular regional level and at the level of causal network connectivity. Collectively, the findings offer important insights into fundamental questions of cerebellar function, and also have implications for the development of methods for enhancing the cognitive abilities of learning and memory. Further studies are needed to systematically address the evolution of learning-driven brain plasticity across time scales, tasks domains and learning approaches.

## Data Availability Statement

The raw data supporting the conclusions of this article will be made available by the authors, without undue reservation.

## Ethics Statement

The studies involving human participants were reviewed and approved by The Smith-Kettlewell Institutional Review Board, Smith-Kettlewell Eye Research Institute. The patients/participants provided their written informed consent to participate in this study.

## Author Contributions

LL conceived and designed the study, led the project, analyses and interpretation, and wrote the manuscript. KM contributed to the data collection, run the analyses and subject management, and contributed to the interpretations. SN performed the MRI scanning and pre-processing. All authors discussed the experiment and the results, and contributed to the manuscript.

## Conflict of Interest

The authors declare that the research was conducted in the absence of any commercial or financial relationships that could be construed as a potential conflict of interest.

## Publisher’s Note

All claims expressed in this article are solely those of the authors and do not necessarily represent those of their affiliated organizations, or those of the publisher, the editors and the reviewers. Any product that may be evaluated in this article, or claim that may be made by its manufacturer, is not guaranteed or endorsed by the publisher.

## Footnotes

1
http://www.diedrichsenlab.org/imaging/suit.htm

2
https://surfer.nmr.mgh.harvard.edu

3
https://web.stanford.edu/group/vista/cgi-bin/wiki/index.php/MrVista

4
http://nist.mni.mcgill.ca/?p=957

5
http://www.diedrichsenlab.org/imaging/mdtb.htm
